# Dosimetric comparison of multiple vs single isocenter technique for linear accelerator‐based stereotactic radiosurgery: The Importance of the six degree couch

**DOI:** 10.1002/acm2.13286

**Published:** 2021-05-21

**Authors:** Dania Amaya, Ashwin Shinde, Christopher Wohlers, Ka Chun Carson Wong, Jennifer Novak, John Neylon, Chunhui Han, An Liu, Savita Dandapani, Scott Glaser

**Affiliations:** ^1^ Department of Radiation Oncology City of Hope National Medical Center Duarte CA USA

**Keywords:** couch, isocenter, radiosurgery, rotational error, six degree

## Abstract

**Purpose:**

**S**ingle isocenter technique (SIT) for linear accelerator‐based stereotactic radiosurgery (SRS) is feasible. However, SIT introduces the potential for rotational error which can lead to geographical miss. Additional planning treatment volume (PTV) margin is required when using SIT. With the six degrees of freedom (6DoF) couch, rotational error can be minimized. We sought to evaluate the effect of the 6DoF couch on the dosimetry of patients with multiple brain metastases treated with SIT.

**Materials and Methods:**

Ten consecutive patients treated with SRS to ≥3 metastases were identified. Original treatments had MIT plans (MITP). The lesions were replanned using SIT. Lesions 5‐10 cm from isocenter had an additional 1mm of margin. Patients were replanned with these additional margins to account for inability to correct rotational error (SITPM). Multiple dosimetric variables and time metrics were evaluated. Dosimetry planning time (DPT) and patient treatment time (PTT) were evaluated. Statistics were calculated using the Wilcoxon signed‐rank test.

**Results:**

A total of 73 brain metastases receiving SRS, to a median of 6 lesions per patient, were identified. MITPs treated 73 lesions with 63 isocenters. On average, MITPs had a 19.2% higher brain V12 than SITPs (*P* = 0.017).

For creation of SITPM, 30 lesions required 1 mm of additional margin, while none required 2 mm of margin. This increased V12 by 47.8% on average per patient (*P* = 0.008) from SITP to SITPM.

DPT was 5.5 hours for SITP, while median for MITP was 12.5 hours (*P* = 0.005) PTT was 30 minutes for SITP, while median for MITP was 144 minutes (*P* = 0.005).

**Conclusions:**

SITPs are comparable to MITPs if rotational error can be corrected with the use of a 6DoF couch. Increasing margin to account for rotational error leads to a nearly 50% increase in V12, which could result in higher rates of radiation necrosis. Time savings are significant using SIT.

## 1 INTRODUCTION

Stereotactic radiosurgery (SRS) is a commonly used method of high dose radiation, pinpointed to areas of radiographically visible disease within the brain. There is increasing interest in the maximum number of brain metastases (BMs) that can be safely treated with SRS rather than whole brain radiation therapy (WBRT), the historical standard. However, the traditional technique of one isocenter per lesion may result in significantly elongated treatment times for an individual patient. Recent studies have demonstrated the feasibility of treating multiple intracranial lesions with a single isocenter.^1^, ^2^, ^3^, ^4^ However, this method has brought up concerns of rotational error, with potential for diminished PTV coverage if not well accounted for 5, 6, 7.

The six degrees of freedom (6DoF) couch is a relatively recent advance that has allowed for improvements in patient reproducibility.^8^ Traditional radiation therapy couches allowed for only longitudinal movements in the x, y, and z axes while rotational errors of yaw, pitch, and roll, were not able to be corrected. The necessity of the 6DoF couch in controlling for rotational error and its effect on dosimetric variables has not been previously studied. We hypothesized that having the 6DoF couch to correct for rotational error would allow for minimal changes to normal organ dosimetry when converting multilesion, multi‐isocenter plans to a multilesion, single isocenter plan. We also hypothesized that adding additional PTV margin to account for that error in the multilesion single isocenter plans (SITP+1) would lead to worsening of normal organ dosimetry. Lastly, we hypothesized that treated with a single isocenter would be more efficient from both a dosimetrist and physicist perspective, while simultaneously reducing how long the patient was on the treatment table.

## 2 MATERIALS AND METHODS

### 2.A Planning and dosimetric factors

We retrospectively identified ten consecutive patients treated with SRS at our institution to ≥3 metastases. The patients were originally planned using MIT, with the exact number of isocenters at the discretion of the treating dosimetrist, physicist, and physician. Contouring and plan formation was done on the Eclipse treatment planning system (Varian, Palo Alto, CA). All plans were created using volumetric arc therapy (VMAT), with goals of covering 95% of all PTVs to 100% of the prescription dose. All patients were treated on a TrueBeam STX linear accelerator with 2.5 mm Micro multileaf collimators (MLCs) as per our institutional standard for all stereotactic treatments. All patients underwent cone beam computed tomography (CBCT) prior to treatment to verify positioning. Original plans were not edited from what each patient had received, and are listed as MIT plans (MITPs). The same planning treatment volumes (PTVs) were replanned at the same prescription dose and normalization using single isocenter technique (SIT), using two coplanar arcs, along with three noncoplanar half arcs at approximately 45, 90, and 135 degrees. Optimal isocenter and noncoplanar arc angles were chosen by the study dosimetrists. These are listed as SIT plans (SITPs).

**Fig. 1:**
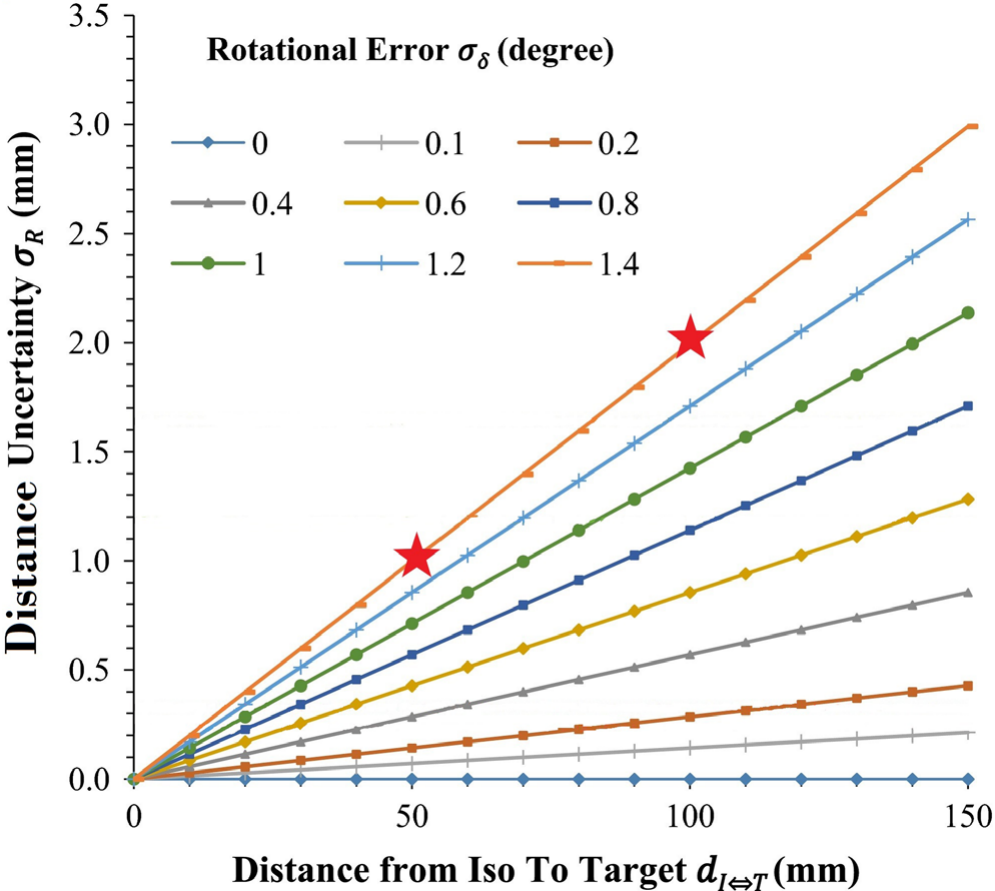
Determination of additional margin necessary based on distance from isocenter to target, based on variable degrees of rotational error. Red stars show that at maximum rotational error, distance from isocenter to target of 5 cm necessitates 1 mm of additional margin, while distance from isocenter to target of 10 cm necessitates 2 mm of additional margin.

SITPs were evaluated to identify PTVs ≥5 cm from the isocenter. The threshold for cut‐off to increase margin, in our model not having the 6DoF couch, was chosen based on a previous publication evaluating distance uncertainty in SRS, as well as a more recent publication from our institution showing concordance.^5^, ^9^ Assuming the max rotational error of 1.4 degrees, a distance from isocenter to target of 5 cm leads to distance uncertainty of 1 mm, while a distance of 10 cm leads to distance uncertainty of 2 mm (Fig. 1). These are listed as SITP with margin (SITPM). The rotational tolerance of a TrueBeam treatment couch, as per manufacturer specifications is ≤0.3 degrees, which leads to a distance uncertainty of ≤0.5 mm at 10 cm from isocenter as per Figure 1. These potential distance uncertainties were not accounted for within our data analyses. Prescription dose and normalization were maintained on all three plans. Prescription dose was between 18 and 21 Gy in 1‐3 fractions. All PTVs had max heterogeneity between 105% and 135%. We evaluated mean brain dose (MBD), volume of brain receiving 4 Gy and 12 Gy in cubic centimeters (V4 and V12, respectively), brainstem max dose, lens max dose, and optic chiasm and nerves max dose as our dosimetric factors of interest across all three sets of plans.

### 2.B Time metrics

The cumulative time required for dosimetry to adequately plan the lesions was calculated as the dosimetry planning time (DPT) for each patient. This included an estimate of 0.5 hours for creation of normal structures. For MITPs, 2 hours per isocenter were estimated; for SITPs, due to increased complexity of the plan and treating multiple lesions simultaneously, 5 hours was estimated as average planning time. The time required for physics verification of the plan to ensure phantom agreement on dosimetry was calculated as the quality assurance time (QAT) for each patient. QAT was estimated as requiring 20 minutes for the first isocenter, and 10 minutes for each additional isocenter. The patient time on table (PTT) for the duration of their treatment was estimated at 20 minutes to allow for cone beam CT verification per isocenter, with a beam on time of 2 minutes per arc that the patient was treated with.

### 2.C Statistical analyses

Statistics were calculated using the paired samples Wilcoxon signed‐rank test.^10^ A threshold of 0.05 for statistical significance was chosen. These analyses were performed for all dosimetric variables comparing MITP to SITP, MITP to SITPM, and SITP to SITPM. DPT, QAT, and PTT were compared between MITP and SITP only, as no changes in time metrics would be expected due to a theoretical increase in PTV margin.

## 3 RESULTS

A total of 10 patients with 73 brain metastases receiving SRS to a median of 6 lesions (range 3‐16) were analyzed. MITPs treated 73 lesions with 63 isocenters while SITPs treated the same 73 lesions with a total of 10 isocenters (one per plan). The prescription dose to the PTV, and normalization, was kept the same for both the MITPs and the SITPs with attempts at keeping hot spots similar as well.

### 3.A Dosimetry

Table 1 displays differences in multiple dosimetric factors comparing MITPs to SITPs. Median brain V12cc was 17% lower with SITPs than MITPs (Range −31.3‐8.6, *P* = 0.017). This corresponded to a 19.2% higher V12 with MITPs than SITPs. Median brain V12 was 9.3cc for MI and 7.3cc for SITPs. Median MBD was 36% higher with SITPs than MITPs (range 0.25‐80.1, *P* = 0.005). However, absolute increases were minimal—median MBD was 125.6 cGy for MITPs and 169.4 cGy for SITPs. A similar increase was seen in lens max dose as well, but there were no statistically significant differences in brain V4, brainstem max dose, or optic structures max dose (Table 1).

**Table 1:** Comparison of single isocenter treatment plans (SITPs) to multi‐isocenter treatment plans (MITPs) across various dosimetric parameters.

Dosimetry Variable	MITP (Median [Range])	SITP (Median [Range])	Percentage change from MITP to SITP (Median [Range])	*P*‐value
Mean Brain Dose (cGy)	125.7 (63.4‐377.1)	169.4 (93.4‐457.6)	35.9 (0.25‐80.1)	0.005
Brain V12 Gy (cc)	9.25 (3.3‐4.4)	7.3 (3.0‐36.0)	‐17 (−31.3‐8.6)	0.017
Brain V4 (cc)	89.7 (26.5‐529.9)	91.3 (21.1‐738.1)	3.2 (−27.5‐39.3)	0.41
Brainstem Max Dose (cGy)	432.1 (8.3‐2520.9)	433.4 (46.9‐2252)	0.2 (−17.4‐472.9)	0.80
Lens Max Dose (cGy)	45.3 (3.6‐24.9)	78 (29‐197)	85.1 (−19.1‐978.04)	0.037
Optic Structures Max Dose (cGy)	322.5 (11.5‐650.1)	324.05 (70.9‐575.4)	10.2 (−40.4‐516.5)	0.65

Table 2 displays differences in multiple dosimetric factors comparing SITPs to SITPMs. There was a 48.1% increase in median V12 from SITPs to SITPMs (*P* = 0.008). Median MBD also increased by 30%. Similar increases were noted in all other dosimetric factors, including brain V4, max dose to brainstem, lens, and optic structures.

**Table 2:** Comparison of single isocenter treatment plans with margin (SITPMs) to single isocenter treatment plans (SITPs) across various dosimetric parameters.

Dosimetry Variable	SITP (Median [Range])	SITPM (Median [Range])	Percentage Change from SITP to SITPM (Median [Range])	*P*‐value
Mean Brain Dose (cGy)	169.4 (93.4‐457.6)	250.1 (117.1‐473.9)	30 (3.6‐73.3)	0.008
Brain V12 Gy (cc)	7.3 (3.0‐36.0)	12.3 (5.9‐38.5)	48.1 (6.9‐ 99.7)	0.008
Brain V4 (cc)	91.3 (21.1‐738.1)	211.65 (41.5‐776.9)	104.4 (5.3‐304.2)	0.008
Brainstem Max Dose (cGy)	433.4 (46.9‐2252)	533 (94.4‐2321.4)	3.1 (−3.4‐80)	0.015
Lens Max Dose (cGy)	78 (29‐197)	107.2 (29.8‐352.3)	28.3 (−1.1‐132.3)	0.013
Optic Structures Max Dose (cGy)	324.05 (70.9‐575.4)	432.3 (73.2‐752.9)	21.1 (3.2‐109.2)	0.008

Finally, Table 3 compares MITPs to SITPMs. While V12 and brainstem max dose are not statistically significantly different, other factors such as MBD, brain V4, lens, and optics are significantly higher with SITPM.

**Table 3:** Comparison of single isocenter treatment plans with margin (SITPMs) to multi‐isocenter treatment plans (MITPs) across various dosimetric parameters.

Dosimetry Variable	MITP (Median [Range])	SITPM (Median [Range])	Percentage Change from MITP to SITPM (Median [Range])	*P*‐value
Mean Brain Dose (cGy)	125.7 (63.4‐377.1)	250.1 (117.1‐473.9)	84.7 (25.7‐148.8)	0.008
Brain V12 Gy (cc)	9.25 (3.3‐4.4)	12. 3 (5.9‐38.5)	8.8 (−11.3‐81.03)	0.173
Brain V4 (cc)	89.7 (26.5‐529.9)	211.65 (41.5‐776.9)	79.7 (23.1‐299.8)	0.008
Brainstem Max Dose (cGy)	432.1 (8.3‐2520.9)	533 (94.4‐2321.4)	2.9 (−7.9‐574.3)	0.21
Lens Max Dose (cGy)	45.3 (3.6‐24.9)	107.2 (29.8‐352.3)	157.4 (44.4‐2017.1)	0.008
Optic Structures Max Dose (cGy)	322.5 (11.5‐650.1)	432.3 (73.2‐752.9)	53.5 (−27.3‐536.5)	0.066

### 3.B Time metrics

Time metrics are summarized in Table 4. DPT was standardized at 5.5 hours for all SITPs, including SITPMs. However, the DPTs for MITPs vary from one another; the median for MITPs was 12.5 hours (range, 6.5‐30.5). DPT for MITPs was significantly higher than for SITPs (*P* = 0.005). QAT was standardized at 20 minutes for SITPs while for MITP, timing was variable, with a median of 70 minutes (40‐160). QAT for MITPs was significantly higher than SITPs (p = 0.005). PTT was 30 minutes for all SITPs. MITPs had variable PTT, with a median of 144 minutes (range, 72‐352). PTT for MITPs was significantly higher than SITPs (*P* = 0.005).

**Table 4:** Comparison of single isocenter treatment plans (SITPs) to multi‐isocenter treatment plans (MITPs) across various time metrics.

	MITP	SITP	*P*‐value
Dosimetry Planning Time (hours)	12.5 (6.5‐30.5)	5.5 (5.5‐5.5)	0.005
Quality Assurance Time (mins)	70 (40‐120)	20 (20‐20)	0.005
Patient Time on Table (mins)	144 (72‐352)	30 (30‐30)	0.005

## 4 DISCUSSION

This study evaluates the dosimetric feasibility of performing single isocentric plans when treating multiple lesions with SRS. Previous studies have also shown the feasibility of this approach.^1^, ^2^, ^3^, ^4^ However, recent publications have demonstrated concerns regarding potential for rotational error and, if unaccounted for, potential for compromised coverage in SRS plans.^5^, ^6^, ^7^

In this study, we accounted for rotational error by either using a six‐degree couch or adding an additional mm or margin for lesions greater than 5cm away. Our study shows that treating with SIT while controlling for rotational error with the 6DoF couch decreases the primary dosimetric parameter critical for SRS plan evaluation, brain V12. However, this benefit is lost when an extra mm of margin is the method of accounting for rotational error, as is required by theoretical modeling as well as verification by our institution’s physics staff.^5^, ^9^ Additionally, not being able to control for rotational error, and thus requiring an extra mm of margin, increases brain V12 by an average of 48% in this series. Brain V12 has been shown to be a consistent predictor of radiation necrosis in multiple series.^11^, ^12^, ^13^ Other dosimetric parameters, such as brain mean and brain V4, may be increased slightly due to transitioning from MIT to SIT. An increase in MBD in SITP may have been driven by a higher V4, potentially as a result of more noncoplanar arcs. While these parameters have not been shown have clinical significance in terms of toxicity, some physicians may be wary of any increase in dosimetric parameters for their SRS patients, and may thus feel that continued use of multi‐isocenter technique is warranted.

The time savings of SIT planning cannot be overstated within this patient cohort. Within our study, dosimetrists required half as much time for planning, physicists required approximately one‐third as much time for quality assurance, and patient time on table was approximately one‐fifth for SIT planning, compared to MIT planning. The time savings, especially for patients, have been corroborated in other series as well.^1^, ^14^, ^15^

The step‐wise increase in feasibility of SRS alone for increasing number of intracranial lesions has been well documented, initially starting with one to three metastases, eventually increasing to ≤10.^16^, ^17^, ^18^ Case reports and retrospective series have also described the feasibility of SRS for patients with greater than ten metastases.^19^, ^20^ SRS in the future will be primarily constrained not by the number of lesions requiring treatment, but rather by the volume of metastatic disease and by the length of time the patient can be on the table undergoing treatment. A SIT plan will be able to minimize that time while still minimizing dose to normal brain.^20^

Limitations of this study include a relatively small sample size without evidence for local control or clinical toxicity outcomes. An unexplained finding of improved V12 in the SITP population may be partially driven by institutional differences in SRS planning, namely the prescription isodose line (IDL). Linac‐based SRS is generally recommended to be prescribed at approximately the 80% IDL, resulting in a max heterogeneity of 125%. While max heterogeneity was reported as a range, we did not look for statistically significant differences in heterogeneity as a potential predictor of V12 across the 73 lesions evaluated. The retrospective nature of this study, and the inherent limitations present are an additional limitation.

We propose prospective validation of these results in future studies, with correlation of both clinical oncologic and toxicity outcomes to identify dosimetric parameters that are clinically significant. Potential future studies should include larger number of patients and quantify a monetary savings associated with decreasing work‐time necessary for dosimetrists/physicists, as well as for radiation therapists at the treatment machine.

In conclusion, we report that accounting for rotational error with a six degrees of freedom couch, when treating multiple (≥3) lesions with a single isocenter technique, results in comparable dosimetry, with significant time savings for the dosimetrist, the physicist, and the patient. However, without a six degrees of freedom couch, the additional margin necessary to account for rotational error results in large changes in critical dosimetric parameters, such as V12, potentially putting patients at higher risk for developing radiation necrosis. We encourage radiation oncologists to consider implementing a single isocenter technique when treating patients with a large number of brain metastases to increase departmental efficiency but to exercise caution in situations where rotational error cannot be appropriately accounted for.

## AUTHOR CONTRIBUTIONS

AS, JN, and SG conceived of the idea. AS, CW, and KCCW performed the computations. DA, AS, JN, CH, AL, and SD verified the analytical methods. DA compiled the results into manuscript form under the guidance of AS, JN, and SG. All authors discussed the results, contributed to the final manuscript, and agree to its contents.

## CONFLICT OF INTEREST

The authors report no conflict of interest.

## Data Availability

The data that support the findings of this study are available from the corresponding author upon reasonable request. Preliminary data for this research was presented as a poster at the ASTRO annual meeting in 2019.
